# Cardiovascular health (“Life’s Essential 8”), risk of depression and anxiety: a prospective cohort study

**DOI:** 10.1038/s44325-024-00023-9

**Published:** 2024-09-13

**Authors:** Shuzhen Liu, Xiangju Hu, Meijie Jiang, Ninghao Huang, Hailun Liang, Ruimao Zheng, Shuyan Wang, Jian Qin, Zhiyong Zhang, Tao Huang, Xu Gao

**Affiliations:** 1 Department of Occupational and Environmental Health Sciences, School of Public Health, Peking University, 100191 Beijing, China; 2 Department for Chronic and Noncommunicable Disease Control and Prevention, Fujian Provincial Center for Disease Control and Prevention, Fuzhou, 350012 Fujian, China; 3 Department of Epidemiology and Biostatistics, School of Public Health, Peking University, 100191 Beijing, China; 4 School of Public Administration and Policy, Renmin University of China, Beijing, China; 5 Department of Anatomy, Histology and Embryology, School of Basic Medical Sciences, Health Science Center, Peking University, 100191 Beijing, China; 6 Neuroscience Research Institute, Peking University, 100191 Beijing, China; 7 Key Laboratory for Neuroscience of Ministry of Education, 100191 Beijing, China; 8 Key Laboratory for Neuroscience of National Health Commission, 100191 Beijing, China; 9 Department of Neurology, Xuanwu Hospital, Capital Medical University, Beijing, China; 10 Department of Environmental and Occupational Health, Guangxi Medical University, 530021 Nanning, China; 11 School of Public Health, Guilin Medical University, 20 Lequn Road, Guilin, Guangxi Zhuang Autonomous Region China; 12 Key Laboratory of Epidemiology of Major Diseases (Peking University), Ministry of Education, 100191 Beijing, China; 13 Center for Healthy Aging, Peking University Health Science Center, 100191 Beijing, China; 14 Peking University Institute of Environmental Medicine, 100191, Beijing, China

**Keywords:** Cardiology, Diseases, Neurology

## Abstract

There is a growing interest in the linkage of cardiovascular health (CVH) with depression/anxiety but the evidence of “Life’s Essential 8” CVH score is scarce. We evaluated the associations of CVH score with risk of incident depression/anxiety among ~0.4 million participants. During follow-up, 17,554 incident events with symptoms of either disorder were recorded. Per 100-point decrease in CVH score was associated with an increased risk of incident either disorder (Hazard ratio [HR] = 1.133, 95% confidence interval [CI]:1.114–1.153), depression (HR = 1.205, 95% CI:1.180–1.231), and anxiety (HR = 1.042, 95% CI:1.017–1.069). Per 100-point decrease in health assessments or health behaviors was associated with an increased risk of incident either disorder (HR_health assessments_ = 1.085, 95% CI: 1.058–1.113, HR_health behaviors_ = 1.217, 95% CI: 1.186–1.250). Poor CVH is a risk factor for the incident late-life depression/anxiety symptoms of middle-aged and older adults, and healthy behaviors could be targeted for the risk assessment and intervention of depression/anxiety.

## Introduction

Depression and anxiety are two common mood disorders that severely endanger human health longevity and have become public health issues due to the rapid population aging^1–3^. Identifying risk factors and finding implementable intervention measures to prevent the occurrence of mood disorders is a public health priority. However, most of the known risk factors for depression and anxiety, such as genetic factors^4^, aging^5^, and childhood adversity^6^, are difficult or impossible to modify. Several modifiable factors, such as air pollution^7^ and lifestyle^8^, have become breakthrough points for preventing the occurrence of anxiety and depression. The extensive body of epidemiologic evidence that cardiovascular diseases (CVDs) are closely related to anxiety and depression^9,10^, and a bidirectional relationship may exist^11^.

In 2010, the American Heart Association defined “cardiovascular health (CVH)” based on seven key health factors and behaviors, including diet, physical activity (PA), smoking, body mass index (BMI), blood lipids, blood pressure (BP), and blood glucose to promote individual and population cardiovascular health (“Life’s Simple 7”)^12^. Over the past decade, CVH was not only widely employed to estimate the risks of cardiovascular events and cognitive health^13–15^, but also the risk of depression. Recently, a prospective community-based cohort of 6,980 participants in France found that better CVH was associated with a lower risk of depressive symptoms over time^16^. It is in line with a longitudinal analysis of ELSA-Brasil, which included 9214 participants and also suggested that poor cardiovascular health tripled depression risk at follow-up in otherwise healthy adults^17^. However, previous studies found that “Life’s Simple 7” was not flawless, the ignorance of inter- and intra-individual heterogeneities and the limited capacity of the seven components were noted^18,19^. Therefore, AHA released a new CVH score named “Life’s Essential 8” in 2022^19^. Components of the new CVH score included updated components for diet, smoking, blood lipids, and blood glucose and additionally included sleep health as the eighth component^19^. We hypothesized that this updated CVH score could act as a better predictor for the CVD-related risks of late-life depression and/or anxiety.

To examine this hypothesis, we conducted a population-based prospective cohort study to evaluate the associations of the “Life’s Essential 8” with the risk of depression and anxiety. We primarily evaluated the associations of CVH with the risk of depression and/or anxiety and secondarily analyzed whether the genetic preposition of depression/anxiety could modify the relationships between CVH and incident depression/anxiety.

## Method

### Study population

UK Biobanks is a large population-based prospective cohort study with approximately 500,000 participants. Details of UKB were described in the previous study^20^. As shown in the flowchart in Supplementary Figure 1, in this study, 447,622 participants with available data on depression/anxiety and CVH scores were included for baseline analysis (analysis 1). The first subset of 388,349 participants who were free of depression or anxiety at baseline was included to evaluate the relationship between CVH and incident depression/anxiety (analysis 2). Among them, a second subset of 129,074 participants who joined a follow-up online mental health survey assessing their mental health including depressive/anxiety symptoms during 2016–2017 was used to evaluate the prospective associations between CVH and the syndromes of depression/anxiety (analysis 3). This subset was further employed in the following symptom analysis. The North West Multicenter Research Ethical Committee has granted the ethics committee approval to the UK Biobank. All participants provided written informed consent at the time of enrollment.

### Assessments of depression and anxiety

Mental health questionnaires and hospital admission records (UK Biobank Data-Fields: 41202 and 1204) were linked to the assessment of depression/anxiety. Detailed assessment was reported in our previous article^7^. Briefly, depression or anxiety was considered positive if the participants’ either record was positive. Considering that anxiety and depression often coexist^21^, either disorder was defined as participants who experienced either anxiety, depression, or both if the participants posed positive records for depression/anxiety. Co-incident symptoms were defined as participants who simultaneously developed depression and anxiety during the follow-up. Participants were assessed by using both the mental health survey and the hospital records in analyses 1 and 2. Participants from the second subset were assessed only by using the online follow-up mental health survey in analysis 3. For hospital records, participants were classified as cases if they had either an ICD-9/10 code of primary or secondary diagnosis for depression (ICD-9: 311; ICD-10: F32–F33) and/or anxiety (ICD-9: 300; ICD-10: F40–F41). For questionnaire screening, at baseline, the Patient Health Questionnaire (PHQ)-4 questionnaire^22^ (consisting of 4 items including a 2-item depression scale and 2-item anxiety scale) was used to evaluate depression and anxiety. At 7-year follow-up, depression, and anxiety symptoms were assessed using PHQ-9 (consists of 9 items)^23^ and generalized anxiety disorder (GAD)-7 (consists of 7 items)^24^ questionnaires. Participants were required to rate, on a four-point Likert scale from 0 (not at all) to 3 (nearly every day), their response to each item. Therefore, the total score of PHQ-4 ranged from 0 to 12 and a score of ≥6 was considered emotional disorder positive. A total score of ≥3 on the depression scale was considered as positive for depression, and a total score of ≥3 on the anxiety scale was considered as anxiety positive^22^. Total scores of PHQ-9 and GAD-7 ranged from 0 to 27 and 0–21, and the PHQ-9 or GAD-7 total score of ≥10 was considered as depression or anxiety symptoms positive, respectively^23,24^. Additionally, any item with a score of ≥1 was considered positive for this symptom.

### Covariates

Age, sex, hypertension, ethnicity, healthy alcohol intake, diabetes, Townsend deprivation index, coronary heart disease (CHD), education, and childhood adversity (an additional covariate for sensitivity analyses) were included as covariates. Ethnicity, healthy alcohol intake, education, history of hypertension, diabetes, and CHD were based on self-reported information and medical records. Ethnicity was categorized into White, Black, Asian, and other. Healthy alcohol intake status was defined as: female: <14 g/day and male: <28 g/day; The medical history of hypertension, CHD, and diabetes was obtained from self-reports and medical records; The Townsend deprivation index, calculated using four variables from the UK Biobank dataset (unemployment, overcrowded household, non–car ownership, and non–home ownership), indicates the level of deprivation, with a higher index indicating a higher level of deprivation; Years of education was classified into <10 years and ≥10 years. Adverse childhood experiences and early Trauma are associated with worse CVH in adults and are risk factors for depression/anxiety across the life-course^25,26^. We examined whether childhood adversity could affect the associations between CVH and incident depression/anxiety, as part of sensitivity analyses. Detailed assessments of childhood adversity were described as supplementary methods.

### Quantification of CVH score

The CVH score of each participant at baseline was calculated, including diet, PA, smoking, sleeping, BMI, blood lipids, blood glucose, and BP according to the AHA algorithm. Each participant’s score scored on a scale of 0–100 points and the total score was calculated by summing the scores for each of the eight components together^19^. The total score was classified into two subgroups including health behaviors (smoking, PA, sleeping, diet) and health assessments (BMI, blood glucose, BP, and blood lipids). Additionally, a 7-item CVH score based on the “Life’s Simple 7” components without the component of sleep health was calculated by summing the scores of diets, PA, smoking, BMI, blood lipids, blood glucose, and BP. Detailed measures of each score were described in supplementary methods.

### Polygenic risk scores

We measured background genetic risk for depression/anxiety disorders using polygenic risk scores (PRS) for depression, anxiety, and a combined depression/anxiety phenotype. Detailed measures of PRS were described in supplementary methods. The best-fitting parameters for the PRS for depression, anxiety, and both disorders were demonstrated (Supplementary Table 1). The resulting PRSs explained of 7.55–8.34% of variance in the odds of depression, and anxiety, either disorder, and the PHQ-4 score at baseline (Supplementary Table 2).

### Statistical analyses

First, the associations of CVH with PHQ-4 scores and the prevalence of depression/anxiety at baseline were examined by linear regression and logistic regression at baseline, respectively (analysis 1). Two models increasingly adjusted for potential covariates: Model 1 controlled for age, and sex. Model 2 increasingly adjusted for hypertension, ethnicity, healthy alcohol intake, diabetes, CHD, Townsend deprivation index, and education. Dose–response curves were assessed by restricted cubic spline regression models^27^ controlling for all potential covariates, with the 25th, 50th, and 75th percentiles of CVH score selected as knots. Additionally, to determine whether the new scores had better predictive power, the associations of the revised 7-item CVH score with the odds of depression and/or anxiety were also examined at baseline as a sensitivity analysis.

Second, the time-to-event analysis between the baseline CVH score and the risk of either disorder, depression, and anxiety at follow-up (the first subset) were tested by using hazard ratio [HR] with 95% confidence intervals [CI] from Cox proportional hazards models (analysis 2). Dose–response relationships related to the risk of the incident, either disorder, depression, or anxiety, were further assessed by the restricted cubic spline regression. The association between CVH score and the co-incidence of depression and anxiety was also evaluated using a competing risk regression model. Subsequently, we tested the prospective associations of the baseline CVH score with the scores of PHQ-9 and GAD-7 and the detailed symptoms of depression/anxiety using linear regression and logistic regression in the second subset of participants with 7-year follow-up data (analysis 3). The aim of this analysis was to evaluate the associations of baseline CVH with the syndromes of depression/anxiety to additionally provide insights into CVH on mental disorders. Similarly, to test whether the new scores had better predictive power, the associations of the revised 7-item CVH score with the risk of depression and/or anxiety were additionally examined as another sensitivity analysis.

We performed three sensitivity analyses to test the robustness of our above findings. First, we examined whether age and sex could interact with CVH in the associations between CVH and the risk of depression/anxiety and the prediction of co-incident depression and anxiety. Then, we tested whether early-life childhood adversity could affect the associations between CVH and incident depression/anxiety among 125,358 participants free of depression/anxiety at baseline with available childhood adversity data retrieved from a follow-up survey. Last, we performed another sensitivity analysis of the second step analysis by excluding participants who were followed for ≤2 years to avoid reversal causality.

Finally, to explore the interactions between the genetic risk of depression/anxiety and CVH score on the incidence of depression/anxiety, we tested the interaction between the CVH score and PRS using the primary Cox proportional hazards model by adding an interaction term of the two factors. In the case that the interaction effect met the criteria for statistical significance, we generated a 16-level categorical variable based on the quartiles of CVH score and PRS. The fourth quartile (Q4) of CVH score and the first quartile (Q1) of PRS were combinedly defined as level 1 for the lowest risk of depression/anxiety, and Q1 of CVH score and Q4 of PRS were combined as the highest risk group accordingly. Associations of the new 16-level variable with the risk of depression/anxiety were evaluated using the Cox model.

R version 4.0.5 (R Development Core Team, Vienna, Austria) and SAS version 9.4 TS1M7 (SAS Institute Inc., Cary, NC, USA) were used for data cleaning and all analyses. *P*-values from the two-sided of <0.05 were considered statistically significant.

## Results

### Characteristics of participants

As shown in Table 1, at baseline, all participants’ average age (mean ± SD) and CVH score were 56.51 ± 8.07 years and 538.72 ± 93.14, 45.0% were men, 49.41% had drunk alcohol, 54.2% were never smokers, and most of them (95.4%) were white. In the hand of major chronic diseases, 27.3% of participants had prevalent hypertension, 5.6% had prevalent CHD, and 5.1% had prevalent diabetes. At baseline, the average PHQ-4 score was 1.61 ± 2.11. About 13.2%, 10.6%, and 6.5% of participants were diagnosed with either disorder, depression, or anxiety based on PHQ-4 scores and hospital records, respectively. In the first subset, 388,349 participants who were free of depression or anxiety at baseline were followed up for a median time of 8.7 years, and the average PHQ-4 score was 0.10 ± 1.18. Among them, 17,554 (4.52%) participants developed either disorder (*N*_depression_ = 11,739; *N*_anxiety_ = 8982). In the second subset with the 7-year follow-up, participants’ s averaged PHQ-7 and GAD-7 scores were 2.28 ± 3.06 and 1.75 ± 2.90, respectively. About 6.6%, 4.5%, and 3.6% of participants demonstrated symptoms meeting the criteria of either disorder, depression, or anxiety based on corresponding scores.

**Table 1:** Characteristics of all participants at baseline and the subgroups of participants

Characteristic^a^	All (*n* = 447,622)	Subgroup 1 (*n* = 388,349)	Subgroup 2 (*n* = 129,074)
Age (years)	56.51 (8.07)	56.78 (8.04)	56.08 (7.73)
*BMI (kg/m*^*2*^*)*			
Underweight or normal weight (<25)	148,548 (33.35%)	131,770 (34.07%)	50,821 (39.46%)
Overweight (25–<30)	189,657 (42.58%)	167,180 (43.22%)	54,003 (41.93%)
Obese (≥30)	10,724 (24.08%)	87,821 (22.71%)	23,983 (18.62%)
Sex (male)	205,745 (45.96%)	181,748 (46.80%)	57,761 (44.75%)
Race (white)	426,996 (95.39%)	373,174 (96.09%)	125,851 (97.50%)
Townsend deprivation index^b^	−1.38 (3.04)	−1.54 (2.95)	−1.80 (2.78)
Years of education (≥10 years)	294,516 (65.80%)	260,198 (67.00%)	102,749 (79.60%)
Healthy alcohol intake (yes)^c^	221,036 (49.41%)	196,649 (50.66%)	69,401 (53.78%)
*Smoking status*			
Current smoker	46,292 (10.37%)	36,169 (9.34%)	8666 (6.72%)
Former smoker	155,804 (34.90%)	136,867 (35.34%)	45,511 (35.31%)
Never smoker	244,271 (54.72%)	214,255 (55.32%)	74,700 (57.96%)
*Major diseases*			
Hypertension^d^	122,489 (27.37%)	102,790 (26.47%)	27,673 (21.44%)
Coronary heart disease^e^	25,256 (5.64%)	19,695 (5.07%)	4275 (3.31%)
Diabetes^f^	22,721 (5.08%)	17,773 (4.58%)	3960 (3.07%)
*Depression/anxiety status at baseline*			
PHQ-4 score	1.61 (2.11)	0.10 (1.18)	
Either disorder	59,273 (13.24%)	17,554 (4.52%)	
Depression	29,005 (6.48%)	11,739 (3.02%)	
Anxiety	47,378 (10.58%)	8982 (2.31%)	
*Depression/anxiety status at 7-year follow-up*			
PHQ-9 score			2.28 (3.06)
GAD-7 score			1.75 (2.90)
Either disorder			8512 (6.59%)
Depression			5808 (4.50%)
Anxiety			4630 (3.59%)
*CVH score at baseline*			
Total score	538.72 (93.14)	543.78 (91.23)	
Health behaviors	292.23 (59.46)	296.03 (57.45)	
Smoking score	78.49 (30.67)	79.54 (29.60)	
Diet score	41.54 (31.91)	42.49 (31.87)	
Sleep score	89.24 (18.71)	90.57 (17.20)	
Physical activity score	82.96 (23.15)	83.42 (23.07)	
Health assessments	246.49 (65.10)	247.23 (64.39)	
BMI score	69.04 (28.52)	69.97 (27.96)	
Blood glucose score	88.26 (20.37)	88.68 (19.92)	
Blood pressure score	41.28 (31.27)	40.76 (31.15)	
Blood lipid score	41.91 (27.29)	47.82 (27.21)	

Note: *BMI* body mass index, *PHQ-4* Patient Health Questionnaire-4 questionnaire, *PHQ-9* Patient Health Questionnaire-9 questionnaire, *GAD-7* General Anxiety Disorder-7 questionnaire.

^a^Mean values (standard deviation) for continuous variables and *n* (%) for categorical variables.

^b^This index is a composite score based on four key variables: unemployment, overcrowded households, non-car ownership, and non-home ownership.

^c^Healthy alcohol intake: male: <28 g/day; female: <14 g/day.

^d^Hypertension diagnosed by the doctor according to self-reported information and/or medical records.

^e^Coronary heart disease diagnosed by the doctor according to self-reported information and/or medical records.

^f^Diabetes diagnosed by the doctor according to self-reported information and/or medical records.

### Association of CVH score with the odds of depression and/or anxiety at baseline

At baseline, participants had higher PHQ-4 scores and were at higher odds of with symptoms of depression, anxiety, or either disorder when they had lower CVH scores (Table 2, Supplementary Fig. 2). After fully adjusting for potential covariates, per 100-point decrease in CVH score was significantly associated with a 0.3-unit (standard error [SE] = 0.0036) increase in the PHQ-4 score, and with a gradually increased odds of either disorder (odds ratio [OR] = 1.458, 95% CI:1.443–1.473), depression (OR = 1.481, 95% CI:1.460–1.501), and anxiety (OR = 1.487, 95% CI:1.470–1.503). Compared to the highest quartile of CVH score, the group of the lowest CVH score quartile showed a 0.7-unit (SE = 0.0092) increase in PHQ-4 score, and the ORs of either disorder, depression, and anxiety were 2.347 (95% CI: 2.284–2.412), 2.414 (95% CI: 2.324–2.507), 2.478 (95% CI: 2.404–2.554), respectively. Compared to the new score, the 7-item CVH score without considering sleep health had a lower predictive power (Supplementary Table 3).

**Table 2:** Associations of the CVH score with the PHQ-4 score and odds of depression and/or anxiety at baseline (Models 1 and 2)^a^

CVH	PHQ-4 score		Either disorder^b^			Depression			Anxiety		
	Coefficients (SE)	*p*-value	*N*_case_/*N*_total_	Odds ratio (95% CI)	*p*-value	*N*_case_/*N*_total_	Odds ratio (95% CI)	*p*-value	*N*_case_/*N*_total_	Odds ratio (95% CI)	*p*-value
*Model 1*											
CVH score (Continuous)	0.4260(0.0034)	**<0.0001**	59,273/447,622	1.613(1.597–1.628)	**<0.0001**	29,005/447,622	1.665(1.644–1.687)	**<0.0001**	47,378/447,622	1.645(1.628–1.663)	**<0.0001**
*CVH score (Quartiles)*											
Q4	Ref		10,606/113,109	Ref		4781/113,109	Ref		8247/113,109	Ref	
Q3	0.2465(0.0088)	**<0.0001**	11,964/109,608	1.382(1.344–1.421)	**<0.0001**	5543/109,608	1.397(1.342–1.454)	**<0.0001**	9472/109,608	1.419(1.376–1.464)	**<0.0001**
Q2	0.4638(0.0088)	**<0.0001**	14,660/112,254	1.794(1.746–1.844)	**<0.0001**	7188/112,254	1.888(1.817-1.962)	**<0.0001**	11,549/112,254	1.831(1.777–1.888)	**<0.0001**
Q1	0.9605(0.0089)	**<0.0001**	22,043/112,651	2.980(2.904–3.058)	**<0.0001**	11,493/11,2651	3.187(3.074–3.304)	**<0.0001**	18,110/112,651	3.145(3.057–3.237)	**<0.0001**
*Model 2*											
CVH score (Continuous)	0.3298(0.0036)	**<0.0001**	59,273/447,622	1.458(1.443–1.473)	**<0.0001**	29,005/447,622	1.481(1.460–1.501)	**<0.0001**	47,378/44,7622	1.487(1.470–1.503)	**<0.0001**
*CVH score (Quartiles)*											
Q4	Ref		10,606/113,109	Ref		4781/113,109	Ref		8247/113,109	Ref	
Q3	0.1930(0.0087)	**<0.0001**	11,964/109,608	1.313(1.276–1.350)	**<0.0001**	5543/109,608	1.311(1.259–1.365)	**<0.0001**	9472/109,608	1.350(1.309–1.394)	**<0.0001**
Q2	0.3459(0.0088)	**<0.0001**	14,660/112,254	1.598(1.554–1.643)	**<0.0001**	7188/112,254	1.643(1.579–1.708)	**<0.0001**	11,549/112,254	1.634(1.584–1.685)	**<0.0001**
Q1	0.7199(0.0092)	**<0.0001**	22,043/112,651	2.347(2.284–2.412)	**<0.0001**	11,493/112,651	2.414(2.324–2.507)	**<0.0001**	18,110/112,651	2.478(2.404–2.554)	**<0.0001**

Note: *PHQ-4* Patient Health Questionnaire-4 questionnaire, associations of CVH with PHQ-4 score were tested with linear regression models, and associations with the odds of either disorders, depression, and anxiety at baseline were tested with logistic regression models. Bold values indicates statistical significant *P* values.

^a^Model 1 adjusted for age and sex; Model 2 additionally adjusted for hypertension, ethnicity, healthy alcohol intake, diabetes, coronary heart disease, Townsend deprivation index, and education.

^b^Either disorder: with depression and/or anxiety, based on PHQ-4 questionnaires and hospital records.

### Association of CVH score with the risk of depression and/or anxiety symptoms during follow-up

As shown in Table 3, for 388,349 participants free of depression/anxiety at baseline, we observed consistent results in crude and fully adjusted models. After fully adjusted, per 100-point decrease in CVH score was associated with an increased risk of incident either disorder (HR = 1.133, 95% CI: 1.114–1.153), depression (HR = 1.205, 95% CI: 1.180–1.231), and anxiety (HR = 1.042, 95% CI: 1.017–1.069), co-incident depression and anxiety (HR = 1.128, 95% CI: 1.083–1.176). Compared to the highest quartile of CVH score, the group of the lowest CVH score quartile was significantly associated with an increased risk of incident events with symptoms of either disorder (HR = 1.341, 95% CI: 1.282–1.403), depression (HR = 1.553, 95% CI: 1.470–1.641), and anxiety (HR = 1.106, 95% CI: 1.038–1.177), co-incident depression and anxiety (HR = 1.337, 95% CI: 1.204–1.485). Compared to the new score, the 7-item score had lower predictive power (Supplementary Table 4).

**Table 3:** Associations of the CVH score at baseline with incident depression and/or anxiety at follow-up (Models 1 and 2)^a^

CVH	Either disorder^b^			Depression			Anxiety			Co-incident depression and anxiety^c^		
	*N*_case_/*N*_total_	Hazard ratio (95% CI)	*p*-value	*N*_case_/*N*_total_	Hazard ratio (95% CI)	*p*-value	*N*_case_/*N*_total_	Hazard ratio (95% CI)	*p*-value	*N*_case_/*N*_total_	Hazard ratio (95% CI)	*p*-value
*Model 1*												
CVH score (Continuous)	17,554/388,349	1.191(1.172–1.211)	**<0.0001**	11739/388,349	1.273(1.248–1.299)	**<0.0001**	8982/338,349	1.089(1.064–1.115)	**<0.0001**	3167/388,349	1.187(1.142–1.234)	**<0.0001**
CVH score (Quartiles)												
Q4	4153/102,503	Ref		2624/102,503	Ref		2337/102,503	Ref		808/102,503	Ref	
Q3	4077/97,644	1.094(1.047–1.142)	**<0.0001**	2647/97,644	1.153(1.092–1.218)	**<0.0001**	2150/97,644	1.017(0.958–1.079)	0.5803	720/97,644	1.064(0.961–1.178)	0.2313
Q2	4452/97,594	1.239(1.186–1.294)	**<0.0001**	2979/97,594	1.355(1.284–1.430)	**<0.0001**	2249/97,594	1.103(1.040–1.171)	**0.0011**	776/97,594	1.217(1.100–1.346)	**0.0001**
Q1	4872/90,608	1.504(1.442–1.570)	**<0.0001**	3489/90,608	1.759(1.670–1.854)	**<0.0001**	2246/90,608	1.225(1.155–1.301)	**<0.0001**	863/90,608	1.507(1.364–1.664)	**<0.0001**
*Model 2*												
CVH score (Continuous)	17,554/388,349	1.133(1.114–1.153)	**<0.0001**	11739/388,349	1.205(1.180–1.231)	**<0.0001**	8982/338,349	1.042(1.017–1.069)	**0.0010**	3167/388,349	1.128(1.083–1.176)	**<0.0001**
CVH score (Quartiles)												
Q4	4153/102,503	Ref		2624/102,503	Ref		2337/102,503	Ref		808/102,503	Ref	
Q3	4077/97,644	1.067(1.021–1.115)	**0.0041**	2647/97,644	1.126(1.066–1.190)	**<0.0001**	2150/97,644	0.987(0.930–1.048)	0.6697	720/97,644	1.031(0.931–1.142)	0.5607
Q2	4452/97,594	1.173(1.122–1.226)	**<0.0001**	2979/97,594	1.281(1.213–1.353)	**<0.0001**	2249/97,594	1.045(0.983–1.110)	0.1571	776/97,594	1.145(1.033–1.269)	**0.0097**
Q1	4872/90,608	1.341(1.282–1.403)	**<0.0001**	3489/90,608	1.553(1.470–1.641)	**<0.0001**	2246/90,608	1.106(1.038–1.177)	**0.0017**	863/90,608	1.337(1.204–1.485)	**<0.0001**

Note: Associations of CVH with the incident either disorder, depression, or anxiety during the follow-up were tested with the Cox proportional hazards model. Bold values indicates statistical significant *P* values.

^a^Model 1 adjusted for age and sex; Model 2 additionally adjusted for hypertension, ethnicity, healthy alcohol intake, diabetes, coronary heart disease, Townsend deprivation index, and education.

^b^Either disorder: with depression and/or anxiety based on 7-year mental health survey and hospital records.

^c^Co-incident depression and anxiety: with depression and anxiety based on 7-year mental health survey and hospital records.

**Fig. 1:**
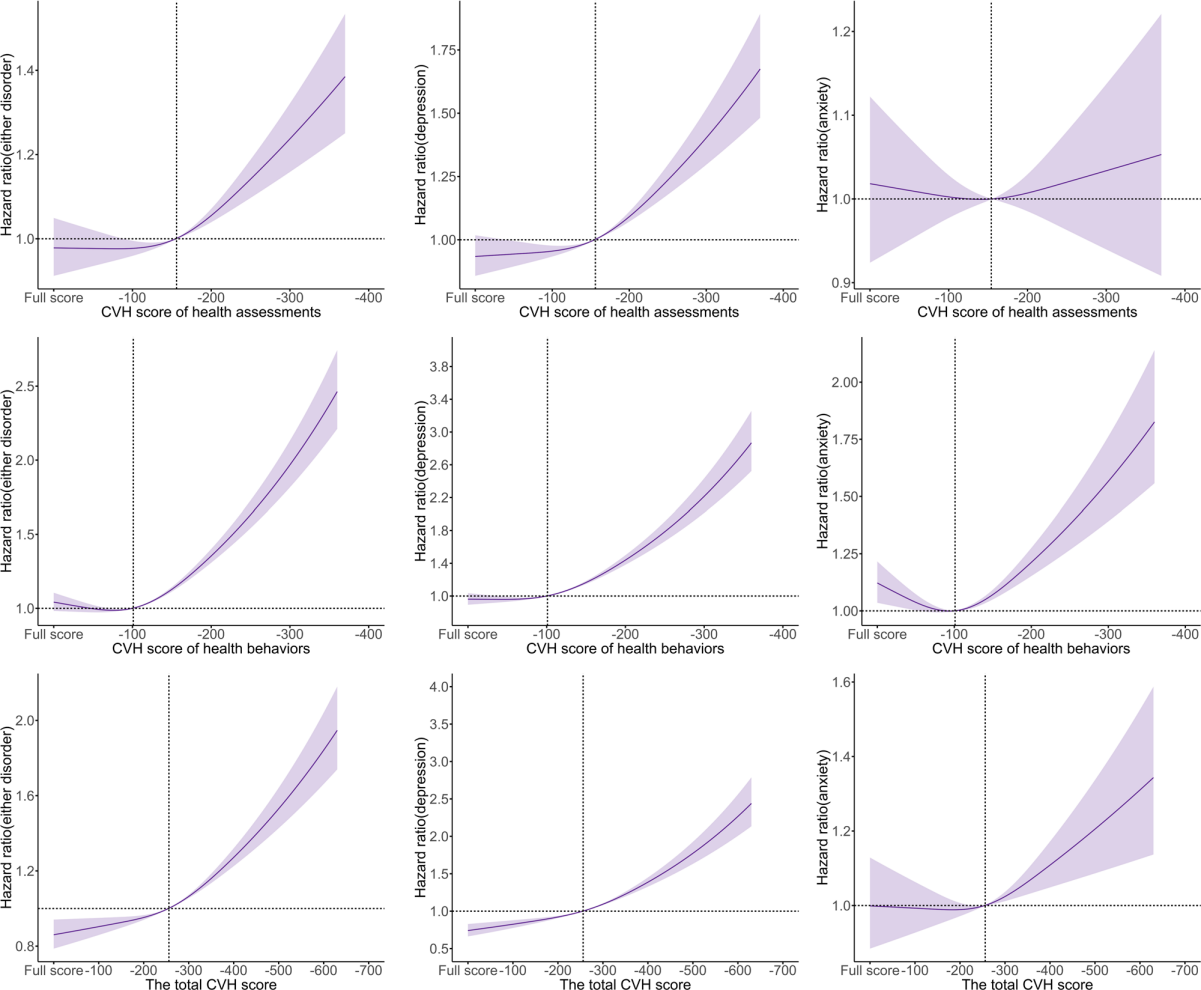
Graphs of the best-fitting models for relationships between CVH score at baseline and depression/anxiety at follow-up for 338,349 participants. Solid line: Point estimation; Shadows: Confidence limits; Dash line: Reference line; The CVH score of health assessments (0–400 points) was calculated based on four components of health assessments (BMI, blood glucose, BP, and blood lipids). The CVH score of health behaviors (0–400 points) was calculated based on four components of human behaviors (smoking, PA, sleeping, and diet). The total CVH score (0–800 points) was calculated based on all components. The restricted cubic spline regression model adjusted for age, sex, ethnicity (White, Black, Asian, and other), healthy alcohol intake(yes/no), years of education (<10 years), Townsend deprivation index, prevalent hypertension, CHD, and diabetes (yes/no). CHD coronary heart disease; Full-score = 400/800 points.

Additionally, significant associations were observed between the risk of all outcomes and components of CVH (Supplementary Table 5). For instance, per 20-point decrease in the smoking score was significantly associated with an increased risk of incident either disorder (HR = 1.106, 95% CI: 1.095–1.116), and per 100-point decrease in health assessments or health behaviors was significantly associated with an increased risk of incident either disorder (HR_health assessments_ = 1.085, 95% CI: 1.058–1.113, HR_health behaviors_ = 1.217, 95% CI: 1.186–1.250). We further observed monotonic increasing trends that the decreased CVH score (including total, health behaviors, and health assessments) was positively associated with a higher risk of all outcomes, except the marginal association between the health assessment score and incident anxiety (Fig. 1).

### Association of CVH score with the odds of depression and/or anxiety symptoms at 7-year follow-up

**Fig. 2:**
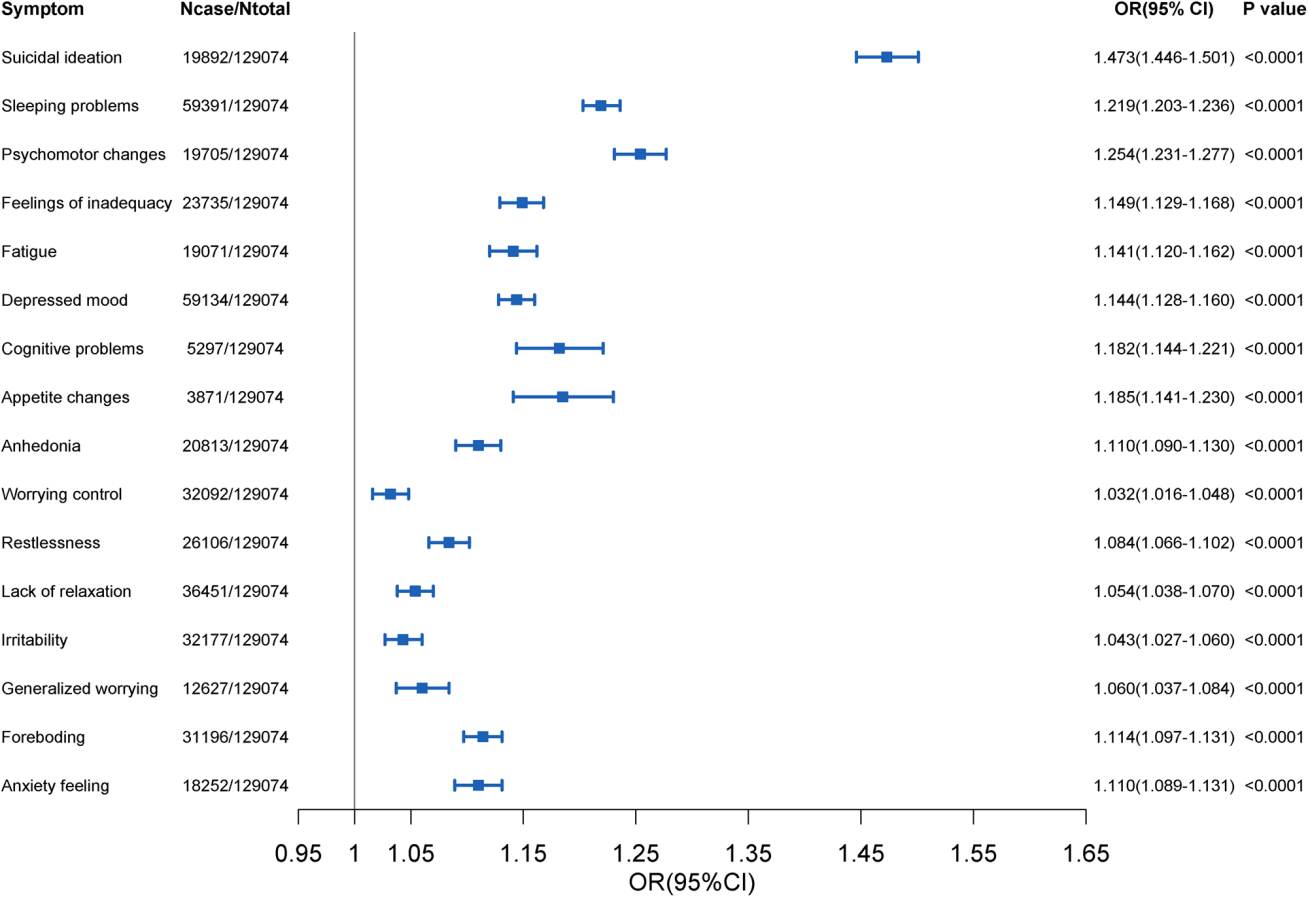
Prospective associations of CVH score at baseline with odds of depression/anxiety at 7-year survey for the 129,074 participants with available data. Models adjusted for age, sex, ethnicity (White, Black, Asian, and other), healthy alcohol intake(yes/no), years of education (<10 years), Townsend deprivation index, prevalent hypertension, CHD, and diabetes (yes/no). Estimates of CVH were demonstrated per 20-point decrease. Dots: Point estimate; Error bar: 95% confidence limits.

Among 120,974 baseline depression/anxiety-free participants with detailed symptoms at the 7-year follow-up, they presented higher PHQ-9 and GDA-7 scores and were more easily with depression, anxiety, either disorder at the 7-year follow-up when they had lower CVH score at baseline (Supplementary Table 6). Per 100-point decrease in CVH score was significantly associated with 0.36 unit (SE = 0.0102) increase in the PHQ-9 score and 0.10 unit (SE = 0.0097) increase in the GDA-7 score and with a gradually increased odds of either disorder (OR = 1.264, 95% CI: 1.231–1.298), depression (OR = 1.371, 95% CI: 1.329–1.414), and anxiety (OR = 1.136, 95% CI:1.097–1.176). Compared to the highest quartile of CVH score, the group of the lowest CVH score quartile was significantly associated with the increased odds of either disorder (OR = 1.637, 95% CI: 1.533–1.749), depression (OR = 1.999, 95% CI: 1.846–2.165), and anxiety (OR = 1.325, 95% CI: 1.208–1.454). CVH score also was associated with each symptom of depression and anxiety (Fig. 2).

Three sensitivity analyses were performed to test the robustness of our above findings. First, we did not observe the interaction of sex and age with CVH in the associations between CVH and the risk of depression/anxiety and the prediction of co-incident depression and anxiety (all *P*_-interaction_ > 0.05, Supplementary Table 7). Then, since we considered whether childhood adversity might affect CVH and incident depression/anxiety, we repeated the analysis of incident depression/anxiety in the subset of participants who were free of depression/anxiety at baseline and who participated in the follow-up online survey that collected reports of childhood adversity (*N* = 125,358). After adjustment for childhood adversity, effect sizes for CVH were similar to effect sizes from our primary analyses of incident depression/anxiety (Supplementary Table 8). Finally, we repeated the analysis in participants who were followed for >2 years to avoid reversing causality. Similarly, we observe a robust association between CVH and the risk of depression/anxiety (Supplementary Table 9).

### Joint effects of CVH score and genetic susceptibility on depression/anxiety

Finally, we found that the interactions between CVH score and PRS were not significant for either disorder (*P*-_interaction_ = 0.5471), depression (*P*-_interaction_ = 0.5248), and anxiety (*P*-_interaction_ = 0.3638). However, the 16-level variable was then generated according to each health outcome, and we observed that compared with the lowest risk level (Q4 of CVH score and Q1 of PRS), participants showed elevated risks of depression/anxiety by the increasing risk levels (Supplementary Fig. 3). For those with the highest risk (Q1 of CVH score and Q4 of PRS), the HRs were 1.313 (95% CI: 1.205–1.431), were1.380 (95% CI: 1.240–1.537) and were 1.255 (95% CI: 1.117–1.411 for either disorder depression and anxiety, respectively (Supplementary Table 10).

## Discussion

In this prospective cohort study, we evaluated the associations of the updated CVH score with prevalent and incident depression and/or anxiety symptoms. Our findings suggested that adults with poorer CVH were more likely to experience depression and/or anxiety in late-life compared with those who had better CVH at baseline. The associations between CVH score and increased risk for depression and either disorder symptoms are mildly modified by the genetic background captured by PRS. To the best of our knowledge, this is the largest prospective cohort study that revealed the association of the redefined “Life’s Essential 8” CVH score with depression/anxiety.

Several previous studies have examined the relationship between the “Life’s Simple 7” CVH and depression^11,17,28^. Our results were consistent with these studies. For instance, a prospective cohort study from ELSA-Brasil found that poor CVH tripled depression risk at follow-up in otherwise healthy adults^17^. Beyond revealing the identical association between CVH and depression in line with these investigations, our study also studied the relationship of CVH score with anxiety and its co-incidence with depression. Although the genetic risk of depression/anxiety did not interact with the CVH score, only a mild modifying effect in the joint analysis, we look forward to a better characterization of depression/anxiety risk with more novel SNPs identified by upcoming GWASs.

More intriguingly, we compared the primary findings with a 7-item CVH score without component sleeping in the sensitivity analyses. We did not observe an association between the 7-item CVH score and the risk of incident anxiety, and the 7-item CVH score had reduced predictive efficacy for prevalent and incident depression and/or anxiety. Consistently, we found that the score of sleeping health appeared to have the most robust association with depression and/or anxiety in all components, suggesting that sleep quality plays a central role in linking CVH with depression/anxiety. This additionally poses another rationale for adding sleep quality to the updated CVH score, as discussed by the AHA Presidential Advisory^19^. Many environmental factors, such as air pollution, can lead to changes in sleep/circadian systems via biological changes including DNA methylation^29^. Evidence indicates that sleep/circadian are closely related to serotonin, a neurotransmitter^30^, which is an important target in the treatment of depression and anxiety^31^. Therefore, it is reasonable to speculate that changes in sleep may lead to the occurrence of anxiety and depression by affecting the levels of certain neurotransmitters.

For other components of CVH, we generally found consistent associations except for BP and diet. In our study, participants with higher BP scores, i.e., better blood pressure, had higher risks of developing anxiety and depression. However, a cross-sectional study showed that participants with ideal BP had a 79% increase in the odds of depression compared to those with poor BP^32^. One of the possible mechanisms for this result is that neurons that could control BP may express neuropeptide Y may play a role in lowering BP and inducing anxiety^33^. Additionally, the associations between depression and/or anxiety and CVH components appeared to be stronger for the CVH behaviors (PA, smoking, sleeping, and diet) than the components of health assessment. A similar trend was also observed in two previous studies^28,34^. For example, Zhang, Z et al. found a graded association between CVH, particularly for health behaviors, and mild and moderate/severe depression among U.S. adults. Altogether, these results suggest that compared with the components of health assessments, CVH-related health behaviors weigh more in leading to depression/anxiety. In addition to the sleep behaviors discussed earlier, regular PA could offer substantial benefits for public mental health^35^. Consistent exercise boosts the production of kynurenine aminotransferase in muscles, leading to a favorable shift in the balance between the neurotoxic kynurenine and the neuroprotective kynurenic acid, thereby reducing the risk of developing mental disorders^36^. Growing evidences also suggested that dietary interventions could serve as a complementary approach to treating mental disorders^37^. A previous review has highlighted various mechanisms through which diet may influence mental health, including modulation of inflammation, oxidative stress, mitochondrial function, gut microbiota, tryptophan–kynurenine metabolism, the hypothalamic–pituitary–adrenal axis, neurogenesis, brain-derived neurotrophic factor levels, epigenetics, and obesity^38^. A large body of evidence has reported that these behavioral changes could potentially trigger anxiety and depression by impacting neurotransmitter levels, the plasticity of neurons, and stress responses, within the brain, as well as altering levels of some inflammatory cytokines^39–41^. In summary, daily behavioral interventions could be an essential approach for preventing mental disorders.

Our study has strengths including the large sample size and the detailed records for constructing the CVH score. We also assessed the background genetic risk for depression/anxiety using PRS leveraging the rich genetic data of UK Biobank. However, we also acknowledge several limitations of this study. Firstly, participants tending to be healthier and wealthier from the UK Biobank do not represent the UK population as a volunteer cohort^42,43^. Relatively healthier and wealthier participants may suffer from less psychosocial pressure. Early-life experiences of mental health problems may also change a person’s tendency to have a bad CVH^44^. Secondly, smoking, PA, diet, sleeping, and alcohol use were self-reported and subject to misclassification and recall biases. Especially the diet score was modified due to the lack of sodium direct relevant data that was instead by salt added to food. We also look forward to more studies in the future using actigraphy measures for a more objective evaluation of CVH metrics, such as sleep and PA. Meanwhile, for the assessment of anxiety and depression, we exclusively relied on questionnaires and medical records. However, given the underestimation and underreporting of mental health issues in older adults is common^45^, more precise and comprehensive diagnoses of mental disorders are recommended in future studies. Thus, the association of CVH with depression and/or anxiety might be over- or under-estimated to some extent. Furthermore, with the limited follow-up, clinically significant changes in CVH components during the follow-up were not comprehensively recorded. This potential bias may lead to the underestimation of the prediction of CVH score on depression/anxiety. Besides, Our analysis did not adjust for medication use that could potentially influence the association between CVH and depression/anxiety, due to the lack of this information. Given our follow-up data was retrieved primarily from links to electronic health records, we believe that most included participants were treated appropriately throughout the study period once diagnosed. Finally, as only about 1/3 of participants joined in the 7-year survey and we observed a larger proportion of participants with depression/anxiety in this subset than the whole participants without depression/anxiety at baseline, the real-world burden of depression and anxiety during the follow-up could be worse than that based on the current information.

In conclusion, we found that adults with worsened cardiovascular health showed an elevated risk of incident events with depression/anxiety symptoms. Under the circumstances of population aging, our findings shed light on the approach that we can promote the health of cardiovascular systems from midlife through healthy behavioral interventions to reduce the late-life incidence of depression/anxiety. This could present on path to reducing the social burden of depression/anxiety.

## Supplementary information

Supplement materials

## Data Availability

Data are available in a public, open-access repository. This research has been conducted using the UK Biobank Resource under Application Number 44430. The UK Biobank data are available on application to the UK Biobank (www.ukbiobank.ac.uk/) with access fees.

## Code availability

Codes of SAS version 9.4 TS1M7 and R version 4.0 for statistical analysis of this study are available upon request from Xu Gao.

## References

[1] Chisholm, D. et al. Scaling-up treatment of depression and anxiety: a global return on investment analysis. *Lancet Psychiatry* **3**, 415–424 (2016). 27083119 10.1016/S2215-0366(16)30024-4

[2] Charlson, F. et al. New WHO prevalence estimates of mental disorders in conflict settings: a systematic review and meta-analysis. *Lancet* **394**, 240–248 (2019). 31200992 10.1016/S0140-6736(19)30934-1 PMC6657025

[3] Whiteford, H. A. et al. Global burden of disease attributable to mental and substance use disorders: findings from the Global Burden of Disease Study 2010. *Lancet* **382**, 1575–1586 (2013). 23993280 10.1016/S0140-6736(13)61611-6

[4] Coombes, B. J. et al. The genetic contribution to the comorbidity of depression and anxiety: a multi-site electronic health records study of almost 178 000 people. *Psychol. Med.* **53**, 7368–7374 (2023). 38078748 10.1017/S0033291723000983 PMC10719682

[5] Gao, X. et al. Accelerated biological aging and risk of depression and anxiety: evidence from 424,299 UK Biobank participants. *Nat. Commun.* **14**, 2277 (2023). 37080981 10.1038/s41467-023-38013-7 PMC10119095

[6] Lai, C. J., Fan, Y., Man, H. Y. & Huang, Y. Childhood adversity and depression in Chinese populations: a multilevel meta-analysis of studies using the Childhood Trauma Questionnaire (CTQ). *Asian J. Psychiatry* **84**, 103582 (2023). 10.1016/j.ajp.2023.103582 37043908

[7] Gao, X., Jiang, M., Huang, N., Guo, X. & Huang, T. Long-term air pollution, genetic susceptibility, and the risk of depression and anxiety: a prospective study in the UK Biobank Cohort. *Environ. Health Perspect.* **131**, 17002 (2023). 36598457 10.1289/EHP10391 PMC9812022

[8] Singh, B. et al. Effectiveness of physical activity interventions for improving depression, anxiety and distress: an overview of systematic reviews. *Br. J. Sports Med.* **57**, 1203–1209 (2023). 36796860 10.1136/bjsports-2022-106195 PMC10579187

[9] Park, K. S. et al. Associations of depression and anxiety with cardiovascular risk among people living with HIV/AIDS in Korea. *Epidemiol. Health* **43**, e2021002 (2021). 33445826 10.4178/epih.e2021002 PMC7952836

[10] Peter, R. S. et al. Long-term trajectories of anxiety and depression in patients with stable coronary heart disease and risk of subsequent cardiovascular events. *Depress. Anxiety* **37**, 784–792 (2020). 32237189 10.1002/da.23011

[11] Ogunmoroti, O. et al. A systematic review of the bidirectional relationship between depressive symptoms and cardiovascular health. *Prev. Med.* **154**, 106891 (2022). 34800472 10.1016/j.ypmed.2021.106891

[12] Lloyd-Jones, D. M. et al. Defining and setting national goals for cardiovascular health promotion and disease reduction: the American Heart Association’s strategic Impact Goal through 2020 and beyond. *Circulation* **121**, 586–613 (2010). 20089546 10.1161/CIRCULATIONAHA.109.192703

[13] Wang, L. et al. Ideal cardiovascular health metric and its change with lifetime risk of cardiovascular diseases: a prospective cohort study. *J. Am. Heart Assoc.* **10**, e022502 (2021). 34755533 10.1161/JAHA.121.022502 PMC8751933

[14] Corlin, L., Short, M. I., Vasan, R. S. & Xanthakis, V. Association of the duration of ideal cardiovascular health through adulthood with cardiometabolic outcomes and mortality in the Framingham Offspring Study. *JAMA Cardiol.* **5**, 549–556 (2020). 32159731 10.1001/jamacardio.2020.0109 PMC7066529

[15] Kulshreshtha, A. et al. Association between cardiovascular health and cognitive performance: a twins study. *J. Alzheimer’s Disease* **71**, 957–968 (2019). 31476151 10.3233/JAD-190217 PMC6918828

[16] van Sloten, T. T. et al. Association of cardiovascular health with risk of clinically relevant depressive symptoms. *JAMA Psychiatry.*10.1001/jamapsychiatry.2022.5056 (2023). 10.1001/jamapsychiatry.2022.5056 PMC9932942 36790776

[17] Brunoni, A. R. et al. Association between ideal cardiovascular health and depression incidence: a longitudinal analysis of ELSA-Brasil. *Acta Psychiatr. Scand.* **140**, 552–562 (2019). 31587258 10.1111/acps.13109

[18] Lloyd-Jones, D. M. et al. Status of cardiovascular health in US adults and children using the American Heart Association’s new “Life’s Essential 8” metrics: prevalence estimates from the National Health and Nutrition Examination Survey (NHANES), 2013 through 2018. *Circulation* **146**, 822–835 (2022). 35766033 10.1161/CIRCULATIONAHA.122.060911

[19] Lloyd-Jones, D. M. et al. Life’s Essential 8: updating and enhancing the American Heart Association’s construct of cardiovascular health: a presidential advisory from the American Heart Association. *Circulation* **146**, e18–e43 (2022). 35766027 10.1161/CIR.0000000000001078 PMC10503546

[20] Sudlow, C. et al. UK biobank: an open access resource for identifying the causes of a wide range of complex diseases of middle and old age. *PLoS Med.* **12**, e1001779 (2015). 25826379 10.1371/journal.pmed.1001779 PMC4380465

[21] Cosci, F. & Fava, G. A. When anxiety and depression coexist: the role of differential diagnosis using clinimetric criteria. *Psychother. Psychosom.* **90**, 308–317 (2021). 34344013 10.1159/000517518

[22] Stanhope, J. Patient Health Questionnaire-4. *Occup. Med. (Oxford, England)* **66**, 760–761 (2016). 10.1093/occmed/kqw165 27994085

[23] Kroenke, K., Spitzer, R. L. & Williams, J. B. The PHQ-9: validity of a brief depression severity measure. *J. Gen. Intern. Med.* **16**, 606–613 (2001). 11556941 10.1046/j.1525-1497.2001.016009606.x PMC1495268

[24] Spitzer, R. L., Kroenke, K., Williams, J. B. & Löwe, B. A brief measure for assessing generalized anxiety disorder: the GAD-7. *Arch. Intern. Med.* **166**, 1092–1097 (2006). 16717171 10.1001/archinte.166.10.1092

[25] Ege, M. A., Messias, E., Thapa, P. B. & Krain, L. P. Adverse childhood experiences and geriatric depression: results from the 2010 BRFSS. *Am. J. Geriatr. Psychiatry* **23**, 110–114 (2015). 25306195 10.1016/j.jagp.2014.08.014 PMC4267899

[26] Islam, S. J. et al. Association between early trauma and ideal cardiovascular health among Black Americans: results from the Morehouse-Emory Cardiovascular (MECA) Center for Health Equity. *Circ. Cardiovasc. Qual. Outcomes* **14**, e007904 (2021). 34380328 10.1161/CIRCOUTCOMES.121.007904 PMC8455434

[27] Desquilbet, L. & Mariotti, F. Dose-response analyses using restricted cubic spline functions in public health research. *Stat. Med.* **29**, 1037–1057 (2010). 20087875 10.1002/sim.3841

[28] Zhang, Z., Jackson, S., Merritt, R., Gillespie, C. & Yang, Q. Association between cardiovascular health metrics and depression among U.S. adults: National Health and Nutrition Examination Survey, 2007–2014. *Ann. Epidemiol.* **31**, 49–56.e42 (2019). 30665827 10.1016/j.annepidem.2018.12.005 PMC10083895

[29] Wang, Y. et al. Short-Term PM(2.5) Exposure and DNA methylation changes of circadian rhythm genes: evidence from two experimental studies. *Environ. Sci. Technol*. 10.1021/acs.est.4c00108 (2024). 10.1021/acs.est.4c00108 38814053

[30] Harvey, A. G., Murray, G., Chandler, R. A. & Soehner, A. Sleep disturbance as transdiagnostic: consideration of neurobiological mechanisms. *Clin. Psychol. review* **31**, 225–235 (2011). 10.1016/j.cpr.2010.04.003 PMC2954256 20471738

[31] Żmudzka, E., Sałaciak, K., Sapa, J. & Pytka, K. Serotonin receptors in depression and anxiety: Insights from animal studies. *Life Sci.* **210**, 106–124 (2018). 30144453 10.1016/j.lfs.2018.08.050

[32] Li, Z. et al. Association between ideal cardiovascular health metrics and depression in Chinese population: a cross-sectional study. *Sci. Rep.* **5**, 11564 (2015). 26176196 10.1038/srep11564 PMC4648472

[33] Michalkiewicz, M., Knestaut, K. M., Bytchkova, E. Y. & Michalkiewicz, T. Hypotension and reduced catecholamines in neuropeptide Y transgenic rats. *Hypertension (Dallas, TX: 1979)* **41**, 1056–1062 (2003). 10.1161/01.HYP.0000066623.64368.4E 12668588

[34] España-Romero, V. et al. A prospective study of ideal cardiovascular health and depressive symptoms. *Psychosomatics* **54**, 525–535 (2013). 24012292 10.1016/j.psym.2013.06.016

[35] Smith, P. J. & Merwin, R. M. The role of exercise in management of mental health disorders: an integrative review. *Annu. Rev. Med.* **72**, 45–62 (2021). 33256493 10.1146/annurev-med-060619-022943 PMC8020774

[36] Pedersen, B. K. Physical activity and muscle-brain crosstalk. *Nat. Rev. Endocrinol.y* **15**, 383–392 (2019). 10.1038/s41574-019-0174-x 30837717

[37] Harris, R. A. et al. Effects of total diet replacement programs on mental well-being: a systematic review with meta-analyses. *Obes. Rev.* **23**, e13465 (2022). 35997170 10.1111/obr.13465 PMC9786773

[38] Marx, W. et al. Diet and depression: exploring the biological mechanisms of action. *Mol. Psychiatry* **26**, 134–150 (2021). 33144709 10.1038/s41380-020-00925-x

[39] Wang, M. et al. Clustered health risk behaviors with comorbid symptoms of anxiety and depression in young adults: moderating role of inflammatory cytokines. *J. Affect. Disord.* **345**, 335–341 (2024). 37898475 10.1016/j.jad.2023.10.139

[40] Ramón-Arbués, E. et al. Health-related behaviors and symptoms of anxiety and depression in Spanish nursing students: an observational study. *Front. Public Health* **11**, 1265775 (2023). 38179570 10.3389/fpubh.2023.1265775 PMC10764461

[41] Menard, C. et al. Social stress induces neurovascular pathology promoting depression. *Nat. Neurosci.* **20**, 1752–1760 (2017). 29184215 10.1038/s41593-017-0010-3 PMC5726568

[42] Fry, A. et al. Comparison of sociodemographic and health-related characteristics of UK Biobank participants with those of the general population. *Am. J. Epidemiol.* **186**, 1026–1034 (2017). 28641372 10.1093/aje/kwx246 PMC5860371

[43] Keyes, K. M. & Westreich, D. UK Biobank, big data, and the consequences of non-representativeness. *Lancet* **393**, 1297 (2019). 30938313 10.1016/S0140-6736(18)33067-8 PMC7825643

[44] Patterson, S. L., Marcus, M., Goetz, M., Vaccarino, V. & Gooding, H. C. Depression and anxiety are associated with cardiovascular health in young adults. *J. Am. Heart Assoc.* **11**, e027610 (2022). 36533593 10.1161/JAHA.122.027610 PMC9798786

[45] Takayanagi, Y. et al. Accuracy of reports of lifetime mental and physical disorders: results from the Baltimore Epidemiological Catchment Area study. *JAMA Psychiatry* **71**, 273–280 (2014). 24402003 10.1001/jamapsychiatry.2013.3579 PMC4135054

