# Supplementary material for: Cardiovascular health (“Life’s Essential 8”), risk of depression and anxiety: a prospective cohort study
Source: NPJ Cardiovasc Health. 2024 Sep 13;1:19. doi: 10.1038/s44325-024-00023-9 (PMC12912395; doi:10.1038/s44325-024-00023-9)
Supplement: Supplementary file 1 — Supplement materials [file 44325_2024_23_MOESM1_ESM.pdf]

Supplement materials

Supplementary methods

Table of contents

Supplementary Table 1 Best-fitting parameters of the polygenic risk scores for depression, anxiety, and either disorder.

Supplementary Table 2 Associations of the odds of depression/anxiety and PHQ-4 score with polygenic risk scores at baseline

Supplementary Table 3 Associations of the 7-itme CVH score with the odds of depression and/or anxiety symptoms at baseline (fully adjusted model)

Supplementary Table 4 Associations of the 7-itme CVH score at baseline with incident depression and/or anxiety symptoms at follow-up (fully adjusted model)

Supplementary Table 5 Associations of components in CVH at baseline with incident depression and/or anxiety symptoms at follow-up (fully adjusted model)

Supplementary Table 6 Associations of CVH score with the PHQ-9 score, GDA-7 score and odds of depression and/or anxiety symptoms at 7-year follow-up (fully adjusted model)

Supplementary Table 7 Interactions between CVH and age/sex in predicting incident depression/anxiety symptoms at follow-up (fully adjusted model)

Supplementary Table 8 Associations of CVH at baseline with incident depression/anxiety symptoms at follow-up among individuals with available follow-up survey data by additionally controlling for childhood adversity (fully adjusted model)

Supplementary Table 9 Associations of CVH at baseline with incident depression/anxiety symptoms at follow-up among individuals with >2 years of follow-up (fully adjusted model)

Supplementary Table 10 Joint associations of CVH score and polygenic risk score with the risks of depression and/or anxiety symptoms during follow-up (fully adjusted model)

Figure of contents

Supplementary Figure 1 Flowchart for the selection of study participants and three subgroups for analysis

Supplementary Figure 2 Graph of the best fitting models for relationships between CVH score and PHQ-4 score for 447,622 participants at baseline.

Supplementary Figure 3 Joint associations of genetic risk and CVH on the risk of either disorder and depression for 338,394 participants at follow-up.

Supplementary methods

1. Detailed measures of each CVH component

(1) Smoking status (UK Biobank Data-Fields: 20116, 2897, 2867 and 1259)**:** CVH score of smoking were determined by self-reported use of cigarettes. Smoking score of CVH who are never and current smoker were calculated as 100 and 0 points, respectively. Former smokers with quitting smoking for at least 5 years were calculated as 75 points. Former smokers who have quit for less than 5 years but at least 1 year were calculated as 50 points. Former smokers with quitting smoking for less than 1 years were calculated as 25 points. All participants who lived with active indoor smoker in home were subtracted 20 points (unless score is 0).

(2) BMI (UK Biobank Data-Fields: 21001)**:** Height and weight were measured in with standardized equipment and techniques. Body mass index (BMI) was calculated by body weight (kg) divided by height squared (m^2). BMI score of CVH who BMI were <25.0 kg/m^2 were calculated as 100 points, ≥40.0 kg/m^2 were calculated as 0 points, 25.0-29.9kg/m^2 and 30.0-34.9kg/m^2, 35.0-39.9kg/m^2 were calculated as 75, 30 and 15 points, respectively.

(3) Physical activity (UK Biobank Data-Fields: 22038, 22039, 894 and 914)**:** Physical activity level was determined using the Metabolic Equivalent Task minutes based on adopted items from the short International Physical Activity Questionnaire. CVH score of physical activity who reported zero minutes/week or more than 150 minutes/week of moderate and greater physical activity were calculated as 0 and 100 points, respectively. People with 120-149, 90-119, 60-89, 30-59, 1-29minutes/week of moderate and greater physical activity were calculated as 90, 80, 60, 40 and 20 points, respectively.

(4) Sleeping (UK Biobank Data-Fields: 1160)**:** CVH score of sleeping were determined based on self-reported average hours of sleep per night. When average hours of sleep per night were <4h, CVH score of sleeping was calculated as 0 point. Scale 4-7h, 5-6h or ≥10h, 6-7h, 7-9h and 9-10h were calculated as 20, 40, 70, 100 and 90 points.

(5) Diet (UK Biobank Data-Fields: fruit [1309, 1319 and 100210], vegetables [1289 and 1299], red and processed meats [1349, 1369, 1379 and 1389], nuts [102430 and 102440], sweetened beverages [100160 and 100170], whole grains [1438, 1448, 1458 and 1468], sodium [1478], low fat [100920, 20106, 102090 and 102810])**:** CVH score of diet was determined by self-reported daily intake of a modified DASH-style eating pattern according to the data availability. After calculating the DASH diet score, the diet score of CVH were categorized as 100 and 0 points for those with a DASH diet score ≥25 and <14 points, respectively. Score 14-16.9, 17-19.9 and 20-24.9 DASH diet score were defined as 25, 50 and 75 points, respectively.

(6) Blood lipids (UK Biobank Data-Fields: 41202 and 41204): CVH score of Blood lipid was determined by non-HDL-cholesterol that calculated by plasma total cholesterol minus HDL-cholesterol. CVH score of Blood lipids who non-HDL-cholesterol were< 130 mg/dL were calculated as 100 points, ≥220 mg/dL were calculated as 0 points, 130-159 mg/dL and 160-189 mg/dL and 190-219 mg/dL were calculated as 60, 40 and 20 points, respectively.

(7) Blood glucose (UK Biobank Data-Fields:30740 and 20750): CVH score of blood glucose were determined by Fasting blood glucose (FBG) and casual hemoglobin A1c(HbA1c). If participants had no history of diabetes and FBG <100 (or HbA1c < 5.7), the score was calculated as 100 points. If participants were not diagnosed as diabetes and 100≤ FBG ≤125(or 5.7≤ HbA1c ≤6.4), the score was calculated as 60 points. If participants were diagnosed as diabetes with HbA1c <7, the score was calculated as 40 points. If participants were diagnosed as diabetes with HbA1c 7.0-7.9, the score was calculated as 30 points. If participants were diagnosed as diabetes with HbA1c 8.0-8.9, the score was calculated as 20 points. The score was calculated as 10 and 0 points when participants were diagnosed as diabetes with HbA1c 9.0-9.9 or HbA1c≥10.0, respectively.

(8) Blood pressure (UK Biobank Data-Fields: 30690, 30760, 6177 and 6153): CVH score of blood pressure were determined by systolic and diastolic blood pressure (SBP and DBP). CVH score of blood pressure

was calculated as 100 points if SBP <120 /DBP <80. The score was calculated as 75 points if SBP=120-129mmHg/DBP <80mmHg, 50 points if SBP=130-139mmHg or DBP=80-89mmHg, 25 points if SBP=140-159mmHg /DBP=90-99mmHg, 0 point if SBP ≥160mmHg or DBP ≥100mmHg. Additionally, the score will be subtracted 20 points if participants were treated.

2. Detailed measures of Polygenic risk scores (PRS)

PRS were computed based on results from the largest published genome-wide association studies (GWASs) of depression and anxiety[1,2]. We also conducted a meta-analysis of the two GWASs using Multi-Trait Analysis of GWAS to produce a GWAS of a combined depression/anxiety phenotype [3]. Detailed information about the genotyping, data imputation, and quality control in the UK Biobank has been reported previously [4,5], Briefly, we excluded SNPs with low minor allele frequency (MAF) (MAF < 1%) or imputation information scores (INFO) (INFO < 0.8). Mismatched, duplicated, and ambiguous SNPs were also excluded. We computed participants PRS by combining the UK Biobank SNP database with summary statistics from the GWAS using the PRSice-2 software [6]. The PRS was adjusted by sex and ten genetic principal components.

3. Assessment of childhood adversity

Studies have shown that childhood adversity might affect CVH and incident depression/anxiety[7,8]. We examined whether childhood adversity could affect the associations between CVH and incident depression/anxiety among 125,358 participants free of depression/anxiety at baseline with available childhood adversity data retrieved from follow-up survey. Five questions from the Childhood Trauma Screener representing physical neglect, emotional neglect, sexual abuse, physical abuse, and emotional abuse were used to assessed Childhood adversity. The Childhood Trauma Screener is a shortened version of the Childhood Trauma Questionnaire and is a cost-efficient, validated, and relatively reliable screening tool in large epidemiological studies[9,10]: (1) felt hated by a family member (emotional abuse, Data-Field: 20487); (2) physically abused by family as a child (physical abuse, Data-Field: 20488); (3) felt loved as a child (emotional neglect, Data-Field: 20489); (4) sexually molested as a child (sexual abuse, Data-Field: 20490); and (5) someone to take to doctor when needed as a child (physical neglect, Data-Field: 20491). For each

question, there are five potential responses included never true, rarely true, sometimes true, often true, and very often true (1-5). If participants answered never true, rarely true, sometimes true, or often true, physical neglect was dichotomized as 1; if participants answered never true, rarely true, or sometimes true, emotional neglect was dichotomized as 1; if participants answered rarely true, sometimes true, often true, and very often true, sexual abuse, physical abuse, and emotional abuse were dichotomized as 1. The summary score of 5 items ranged from 0 to 5, with a higher score denoting more childhood adversities

References

1 Als, T. D. *et al.* Depression pathophysiology, risk prediction of recurrence and comorbid psychiatric disorders using genome-wide analyses. *Nature medicine* **29**, 1832-1844, doi:10.1038/s41591-023-02352-1 (2023).

2 Otowa, T. *et al.* Meta-analysis of genome-wide association studies of anxiety disorders. *Molecular psychiatry* **21**, 1485, doi:10.1038/mp.2016.11 (2016).

3 Willer, C. J., Li, Y. & Abecasis, G. R. METAL: fast and efficient meta-analysis of genomewide association scans. *Bioinformatics* **26**, 2190-2191, doi:10.1093/bioinformatics/btq340 (2010).

4 Sudlow, C. *et al.* UK biobank: an open access resource for identifying the causes of a wide range of complex diseases of middle and old age. *PLoS medicine* **12**, e1001779, doi:10.1371/journal.pmed.1001779 (2015).

5 Bycroft, C. F., C.; Petkova, D.; Band, G.; Elliott, L. T.; Sharp, K.; Motyer, A.; Vukcevic, D.; Delaneau, O.; & O'Connell, J. e. a. Genome-wide genetic data on ~500,000 UK Biobank participants. *bioRxiv*, 166298, doi:10.1101/166298 (2017).

6 Choi, S. W. & O'Reilly, P. F. PRSice-2: Polygenic Risk Score software for biobank-scale data. *GigaScience* **8**, doi:10.1093/gigascience/giz082 (2019).

7 Ege, M. A., Messias, E., Thapa, P. B. & Krain, L. P. Adverse childhood experiences and geriatric depression: results from the 2010 BRFSS. *The American journal of geriatric psychiatry : official journal of the American Association for Geriatric Psychiatry* **23**, 110-114, doi:10.1016/j.jagp.2014.08.014 (2015).

8 Islam, S. J. *et al.* Association Between Early Trauma and Ideal Cardiovascular Health Among Black Americans: Results From the Morehouse-Emory Cardiovascular (MECA) Center for Health Equity. *Circulation. Cardiovascular quality and outcomes* **14**, e007904, doi:10.1161/circoutcomes.121.007904 (2021).

9 Grabe, H. J. *et al.* [A brief instrument for the assessment of childhood abuse and neglect: the childhood trauma screener (CTS)]. *Psychiatrische Praxis* **39**, 109-115, doi:10.1055/s-0031-1298984 (2012).

10 Glaesmer, H. & Brähler, E. [Commentary to Grabe HJ et al.: a brief instrument for the assessment of childhood abuse and neglect: the Childhood Trauma Screener (CTS)]. *Psychiatrische Praxis* **40**, 21-22, doi:10.1055/s-0032-1327226 (2013).

Supplementary Table 1 Best-fitting parameters of the polygenic risk scores for depression, anxiety, and either disorder.

Outcomes	Full R-squares	Numbers of selected SNPs
Depression	0.041032	- 5510331
Anxiety	0.0123399	14193

Supplementary Table 2 Associations of the odds of depression/anxiety and PHQ-4 score with polygenic risk scores at baseline [a]

Trait	Coefficients	SE	R-squares (variance explained)	*p*-value
Prevalent depression	0.0375	0.0066	0.0755	**<0.0001**
Prevalent anxiety	0.0356	0.0053	0.0809	**<0.0001**
Prevalent either disorder	0.0110	0.0048	0.0834	**0.0226**
PHQ-4 score	0.0440	0.0031	0.0789	**<0.0001**

a: Associations of depression, anxiety, and either disorder at baseline with polygenic risk scores were tested by logistic regression models; association of PHQ-4 score with polygenic risk score of depression and/or anxiety were tested by linear regression models. Models were adjusted for age, sex, ethnicity, BMI, smoking status, healthy alcohol intake, healthy physical activity, hypertension, diabetes, coronary heart disease, Townsend deprivation index and education.

Note: PHQ-4, Patient Health Questionnaire-4 questionnaire; Associations of polygenic risk scores with PHQ-4 score were tested with linear regression models and associations with the odds of either disorders, depression, and anxiety at baseline were tested with logistic regression models.

Supplementary Table 3 Associations of the 7-itme CVH score with the odds of depression and/or anxiety symptoms at baseline (fully adjusted model) [a]

CVH	Either disorder [b]		Depression		Anxiety	
	**Odds ratio (95% CI)**	***p*-value**	**Odds ratio (95% CI)**	***p*-value**	**Odds ratio (95% CI)**	***p*-value**
**7-item score [c]**	1.327(1.313-1.341)	**<0.0001**	1.356(1.336-1.375)	**<0.0001**	1.342(1.327-1.358)	**<0.0001**

a: Model adjusted for age, sex, hypertension, ethnicity, healthy alcohol intake, diabetes, coronary heart disease, Townsend deprivation index and education;

b: Either disorder: with depression and/or anxiety, based on PHQ-4 questionnaires and hospital records;

c: The score only based on based on the "Life's Simple 7" components without the component of sleep health was calculated by summing the scores of diets, PA, smoking, BMI, blood lipids, blood glucose, and BP.

Note: Associations of CVH with the odds of either disorders, depression, and anxiety at baseline were tested with logistic regression models.

Supplementary Table 4 Associations of the 7-itme CVH score at baseline with incident depression and/or anxiety symptoms at follow-up (fully adjusted model) [a]

CVH	Either disorder [c]		Depression		Anxiety		Co-incident depression and anxiety [d]	
	Hazard ratio (95% CI)	*p*-value	Hazard ratio (95% CI)	*p*-value	Hazard ratio (95% CI)	*p*-value	Hazard ratio (95% CI)	*p*-value
**7-item score [d]**	1.089(1.070-1.109)	**<0.0001**	1.152(1.127-1.178)	**<0.0001**	1.004(0.979-1.030)	0.7456	1.068(1.023-1.114)	**0.0024**

a: Model additionally adjusted for sex, hypertension, ethnicity, healthy alcohol intake, diabetes, coronary heart disease, Townsend deprivation index and education;

b: Either disorder: with depression and/or anxiety based on 7-year mental health survey and hospital records;

c: Co-incident depression and anxiety: with depression and anxiety based on 7-year mental health survey and hospital records;

d: The score only based on based on the "Life's Simple 7" components without the component of sleep health was calculated by summing the scores of diets, PA, smoking, BMI, blood lipids, blood glucose, and BP.

Note: Associations of CVH with the incident either disorder, depression, and anxiety during the follow-up were tested with Cox proportional hazards models.

Supplementary Table 5 Associations of components in CVH at baseline with incident depression and/or anxiety symptoms at follow-up (fully adjusted model) [a]

Components	Incident either disorder [b]		Incident depression		Incident anxiety	
	Hazard ratio (95% CI)	*p*-value	Hazard ratio (95% CI)	*p*-value	Hazard ratio (95% CI)	*p*-value
Blood lipids	1.020(1.009-1.032)	**0.0005**	1.031(1.017-1.045)	**<0.0001**	1.004(0.988-1.020)	0.6436
Blood pressure	0.962(0.952-0.973)	**<0.0001**	0.952(0.940-0.965)	**<0.0001**	0.973(0.959-0.988)	**0.0003**
Blood glucose	1.032(1.012-1.052)	**0.0013**	1.050(1.026-1.075)	**<0.0001**	1.003(0.976-1.030)	0.8482
BMI	1.086(1.075-1.098)	**<0.0001**	1.138(1.124-1.153)	**<0.0001**	1.026(1.011-1.042)	**0.0008**
Health assessments	1.085(1.058-1.113)	**<0.0001**	1.152(1.116-1.188)	**<0.0001**	1.1006(0.971-1.043)	0.7481
Physical activity	1.019(1.006-1.033)	**0.0036**	1.025(1.009-1.041)	**0.0018**	0.994(0.976-1.013)	0.5417
Sleeping	1.206(1.188-1.224)	**<0.0001**	1.249(1.227-1.271)	**<0.0001**	1.170(1.146-1.196)	**<0.0001**
Diet	0.950(0.941-0.959)	**<0.0001**	0.959(0.947-0.970)	**<0.0001**	0.936(0.923-0.948)	**<0.0001**
Smoking	1.106(1.095-1.116)	**<0.0001**	1.128(1.115-1.140)	**<0.0001**	1.076(1.062-1.090)	**<0.0001**
Health behaviors	1.217(1.186-1.250)	**<0.0001**	1.311(1.270-1.353)	**<0.0001**	1.093(1.053-1.135)	**<0.0001**
Total	1.133(1.114-1.153)	**<0.0001**	1.205(1.180-1.231)	**<0.0001**	1.042(1.017-1.069)	**0.0010**

a: Model adjusted for age, sex, hypertension, ethnicity, healthy alcohol intake, diabetes, coronary heart disease, Townsend deprivation index and education;

b: Either disorder: with depression and/or anxiety based on 7-year mental health survey and hospital records.

Note: Associations of components in CVH at baseline with the incident either disorder, depression, and anxiety during the follow-up were tested with Cox proportional hazards models.

Supplementary Table 6 Associations of CVH score with the PHQ-9 score, GDA-7 score and odds of depression and/or anxiety symptoms at 7-year follow-up (fully adjusted model) [a]

CVH	PHQ-9-score		GDA-7 score		Either disorder [b]			Depression			Anxiety		
	**Coefficients (SE)**	***p*-value**	**Coefficients (SE)**	***p*-value**	**Ncase/Ntotal**	**Odds ratio (95% CI)**	***p*-value**	**Ncase/Ntotal**	**Odds ratio (95% CI)**	***p*-value**	**Ncase/Ntotal**	**Odds ratio (95% CI)**	***p*-value**
CVH score (Continuous)	0.3642(0.0102)	**<0.0001**	0.1022(0.0097)	**<0.0001**	8515/120974	1.264(1.231-1.298)	**<0.0001**	5808/129074	1.371(1.329-1.414)	**<0.0001**	4630/129074	1.136(1.097-1.176)	**<0.0001**
CVH score (Quartiles)													
Q4	Ref		Ref		1925/32815	Ref		1206/32815	Ref		1145/32815	Ref	
Q3	0.1970(0.0240)	**<0.0001**	0.0844(0.0230)	**0.0002**	1810/31110	1.125(1.052-1.203)	**0.0006**	1166/31110	1.183(1.089-1.286)	**<0.0001**	1057/31110	1.098(1.006-1.198)	**0.0373**
Q2	0.3877(0.0242)	**<0.0001**	0.1122(0.0231)	**<0.0001**	2137/32881	1.309(1.225-1.398)	**<0.0001**	1450/32881	1.456(1.343-1.579)	**<0.0001**	1161/32881	1.187(1.086-1.298)	**0.0002**
Q1	0.7756(0.0251)	**<0.0001**	0.2165(0.0240)	**<0.0001**	2640/32268	1.637(1.533-1.749)	**<0.0001**	1986/32268	1.999(1.846-2.165)	**<0.0001**	1267/32268	1.325(1.208-1.454)	**<0.0001**

a: Model adjusted for age, sex, hypertension, ethnicity, healthy alcohol intake, diabetes, coronary heart disease, Townsend deprivation index and education;

b: Either disorder: with depression and/or anxiety based on 7-year mental health survey and hospital records;

Note: PHQ-9, Patient Health Questionnaire-9 questionnaire; GAD-7, General Anxiety Disorder-7 questionnaire; Associations of CVH with PHQ-9 and GDA-7 score were tested with linear regression models and associations with the odds of either disorders, depression, and anxiety at 7-year follow-up were tested with logistic regression models.

Supplementary Table 7 Interactions between CVH and age/sex in predicting incident depression/anxiety symptoms at follow-up (fully adjusted model) [a]

$P_{\text{-interaction}}$	Either disorder [b]	Incident depression	Incident anxiety	Co-incident depression and anxiety
Sex	0.1596	0.7886	0.2028	0.9168
Age (continuous variable)	0.2197	0.4252	0.6562	0.4840

a: Model adjusted for age, sex, hypertension, ethnicity, healthy alcohol intake, diabetes, coronary heart disease, Townsend deprivation index and education;

b: Either disorder: with depression and/or anxiety based on 7-year mental health survey and hospital records.

Supplementary Table 8 Associations of CVH at baseline with incident depression/anxiety symptoms at follow-up among individuals with available follow-up survey data by additionally controlling for childhood adversity (fully adjusted model) [a]

CVH	Either disorder [b]			Incident depression			Incident anxiety		
	**Ncase/Ntotal**	**Hazard ratio (95% CI)**	**p-value**	**Ncase/Ntotal**	**Hazard ratio (95% CI)**	**p-value**	**Ncase/Ntotal**	**Hazard ratio (95% CI)**	**p-value**
CVH score (Continuous)	8230/125358	1.210(1.179-1.241)	**<0.0001**	5616/125358	1.305(1.266-1.345)	**<0.0001**	4474/125358	1.098(1.061-1.137)	**<0.0001**
CVH score (Quartiles)									
Q4	1880/31976	Ref		1178/31976	Ref		1119/31976	Ref	
Q3	1746/30239	1.089(1.019-1.163)	**0.0017**	1124/30239	1.140(1.049-1.239)	**0.0019**	1015/30239	1.055(0.968-1.150)	0.2266
Q2	2050/31822	1.234(1.157-1.317)	**<0.0001**	1395/31822	1.368(1.263-1.482)	**<0.0001**	1109/31822	1.106(1.015-1.205)	**0.0218**
Q1	2554/31321	1.494(1.401-1.594)	**<0.0001**	1919/31321	1.801(1.665-1.948)	**<0.0001**	1231/31321	1.201(1.101-1.311)	**<0.0001**

a: Model adjusted for age, sex, hypertension, ethnicity, healthy alcohol intake, diabetes, coronary heart disease, education, Townsend deprivation index and childhood adversity;

b: Either disorder: with depression and/or anxiety based on 7-year mental health survey and hospital records;

Note: Associations of components in CVH at baseline with the incident either disorder, depression, and anxiety during the follow-up were tested with Cox proportional hazards models.

Supplementary Table 9 Associations of CVH at baseline with incident depression/anxiety symptoms at follow-up among individuals with >2 years of follow-up (fully adjusted model) [a]

CVH	Either disorder [b]			Incident depression			Incident anxiety		
	**Ncase/Ntotal**	**Hazard ratio (95% CI)**	**p-value**	**Ncase/Ntotal**	**Hazard ratio (95% CI)**	**p-value**	**Ncase/Ntotal**	**Hazard ratio (95% CI)**	**p-value**
CVH score (Continuous)	17499/386684	1.132(1.112-1.152)	**<0.0001**	11700/386684	1.204(1.178-1.230)	**<0.0001**	8963/386684	1.041(1.016-1.067)	**0.0013**
CVH score (Quartiles)									
Q4	3918/96671	ref		2482/96671	ref		2195/96671	ref	
Q3	3948/94760	1.069(1.022-1.118)	**0.0037**	2554/94760	1.121(1.060-1.186)	**<0.0001**	2099/94760	1.002(0.943-1.065)	0.9476
Q2	4462/98582	1.166(1.115-1.219)	**<0.0001**	2961/98582	1.260(1.192-1.332)	**<0.0001**	2283/98582	1.055(0.992-1.121)	0.0870
Q1	5171/96671	1.336(1.277-1.397)	**<0.0001**	3703/96671	1.545(1.462-1.633)	**<0.0001**	2386/96671	1.106 (1.039-1.178)	**0.0017**

a: Model adjusted for age, sex, hypertension, ethnicity, healthy alcohol intake, diabetes, coronary heart disease, Townsend deprivation index and education;

b: Either disorder: with depression and/or anxiety based on 7-year mental health survey and hospital records;

Note: Associations of components in CVH at baseline with the incident either disorder, depression, and anxiety during the follow-up were tested with Cox proportional hazards models.

Supplementary Table 10 Joint associations of CVH score and polygenic risk score with the risks of depression and/or anxiety symptoms during follow-up (fully adjusted model) [a]

Levels (quartile of CVH score & quartile of PRs)	Incident depression		Incident anxiety		Incident either disorder	
	**HR (95%CI)**	***p*-values**	**HR (95%CI)**	***p*-values**	**HR (95%CI)**	***p*-values**
Level 1 (Q4&Q1)	Ref		Ref		Ref	
Level 2 (Q4&Q2)	0.919(0.822-1.029)	0.1429	0.948(0.839-1.071)	0.3910	1.029(0.941-1.124)	0.5341
Level 3 (Q4&Q3)	0.910(0.813-1.018)	0.0990	1.024(0.908-1.154)	0.7013	0.968(0.885-1.058)	0.4723
Level 4 (Q4&Q4)	**0.876(0.782-0.982)**	**0.0229**	1.065(0.946-1.199)	0.2955	0.977(0.893-1.068)	0.6061
Level 5 (Q3&Q1)	**1.136(1.016-1.271)**	**0.0257**	0.939(0.829-1.064)	0.3235	**1.098(1.005-1.200)**	**0.0387**
Level 6 (Q3&Q2)	1.045(0.935-1.170)	0.4377	0.975(0.862-1.104)	0.6930	**1.123(1.027-1.227)**	**0.0109**
Level 7 (Q3&Q3)	1.059(0.947-1.184)	0.3153	1.030(0.911-1.163)	0.6377	1.015(0.927-1.111)	0.7550
Level 8 (Q3&Q4)	0.933(0.831-1.048)	0.2444	1.111(0.986-1.252)	0.0833	1.025(0.936-1.122)	0.5942
Level 9 (Q2&Q1)	**1.286(1.154-1.435)**	**<0.0001**	1.052(0.932-1.187)	0.4129	**1.165(1.068-1.272)**	**0.0006**
Level 10 (Q2&Q2)	**1.163(1.043-1.297)**	**0.0068**	1.092(0.969-1.231)	0.1504	**1.136(1.040-1.241)**	**0.0048**
Level 11 (Q2&Q3)	**1.150(1.031-1.283)**	**0.0122**	1.028(0.911-1.161)	0.6520	**1.207(1.107-1.317)**	**<0.0001**
Level 12 (Q2&Q4)	**1.128(1.010-1.261)**	**0.0327**	1.107(0.983-1.248)	0.0947	**1.151(1.055-1.257)**	**0.0016**
Level 13 (Q1&Q1)	**1.575(1.419-1.750)**	**<0.0001**	1.073(0.950-1.212)	0.2599	**1.391(1.278-1.514)**	**<0.0001**
Level 14 (Q1&Q2)	**1.503(1.354-1.669)**	**<0.0001**	1.088(0.964-1.229)	0.1707	**1.308(1.199-1.426)**	**<0.0001**
Level 15 (Q1&Q3)	**1.406(1.265-1.563)**	**<0.0001**	1.077(0.954-1.216)	0.2339	**1.337(1.227-1.457)**	**<0.0001**
Level 16 (Q1&Q4)	**1.380(1.240-1.537)**	**<0.0001**	**1.255(1.117-1.411)**	**0.0001**	**1.313(1.205-1.431)**	**<0.0001**

a: Model additionally adjusted for age, sex, hypertension, ethnicity, healthy alcohol intake, diabetes, coronary heart disease, Townsend deprivation index and education;

b: Either disorder: with depression and/or anxiety based on 7-year mental health survey and hospital records.

Note: Joint associations of CVH score and polygenic risk score with the incident either disorder and depression during the follow-up were tested with Cox proportional hazards models

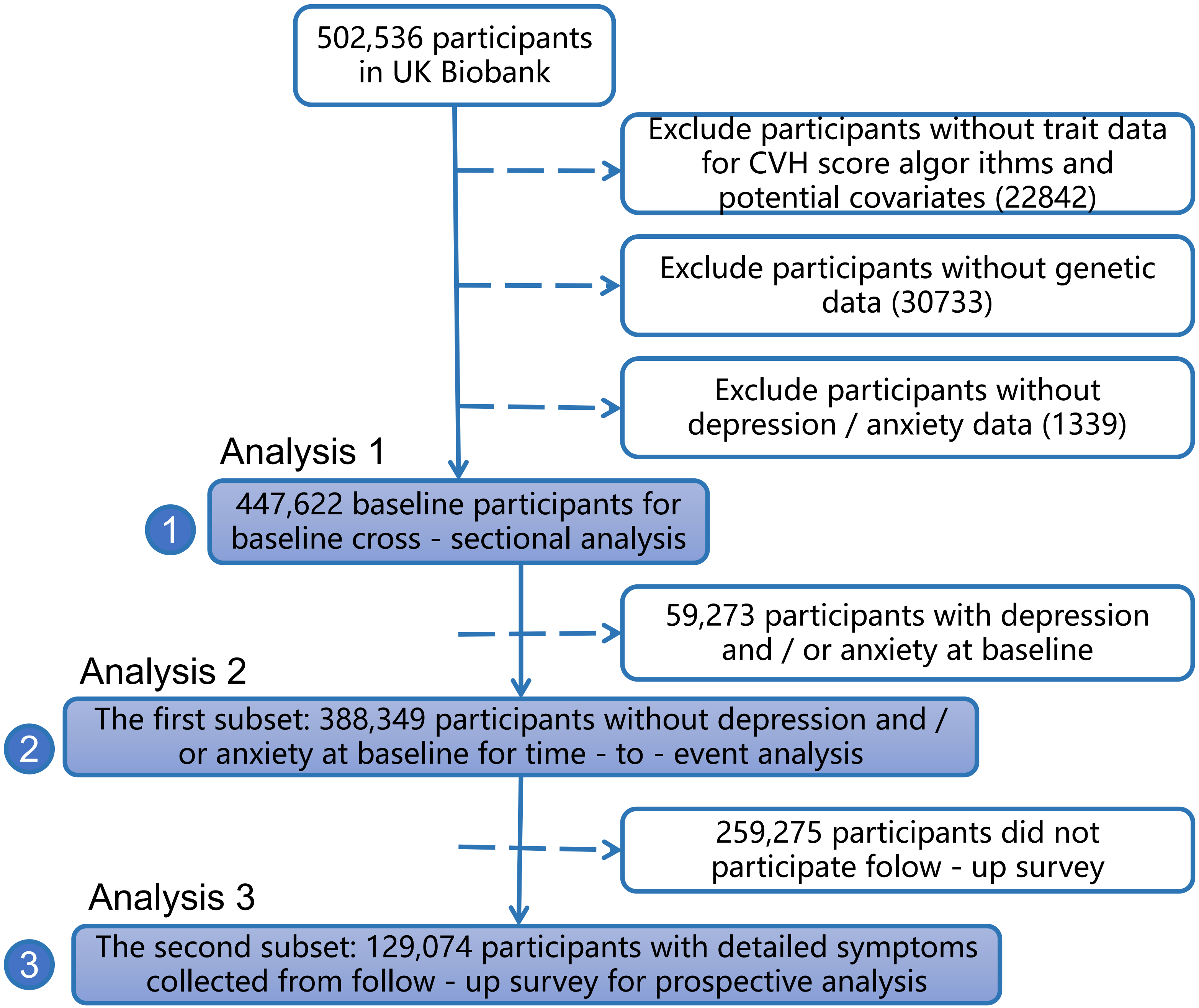

502,536 participants in UK Biobank
Exclude participants without trait data for CVH score algor ithms and potential covariates (22842)
Exclude participants without genetic data (30733)
Exclude participants without depression / anxiety data (1339)
Analysis 1
1
447,622 baseline participants for baseline cross - sectional analysis
59,273 participants with depression and / or anxiety at baseline
Analysis 2
2
The first subset: 388,349 participants without depression and / or anxiety at baseline for time - to - event analysis
259,275 participants did not participate folow - up survey
Analysis 3
3
The second subset: 129,074 participants with detailed symptoms collected from follow - up survey for prospective analysis

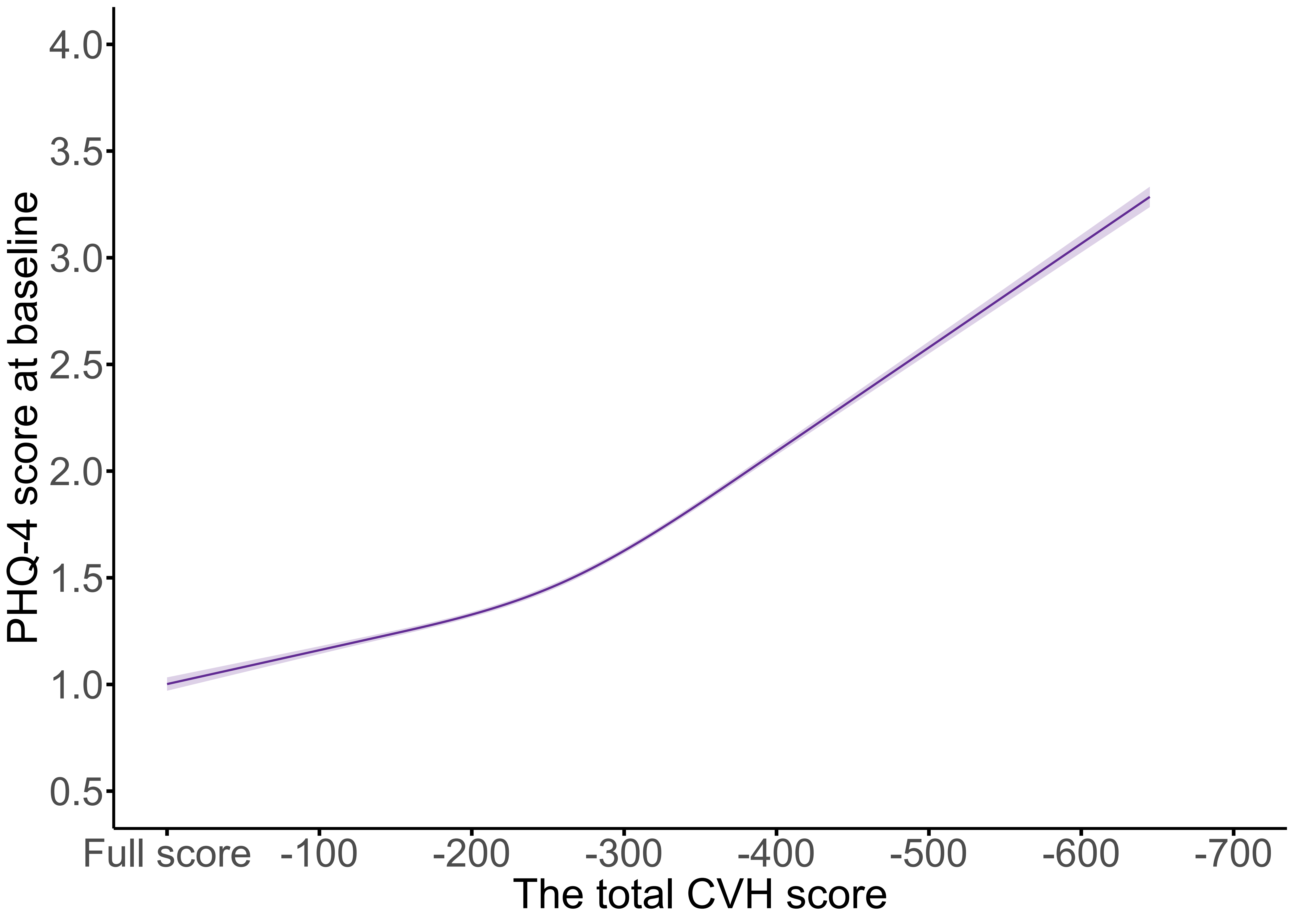
PHQ-4 score at baseline
4.0
3.5
3.0
2.5
2.0
1.5
1.0
0.5
Full score
-100
-200
-300
-400
-500
-600
-700
The total CVH score

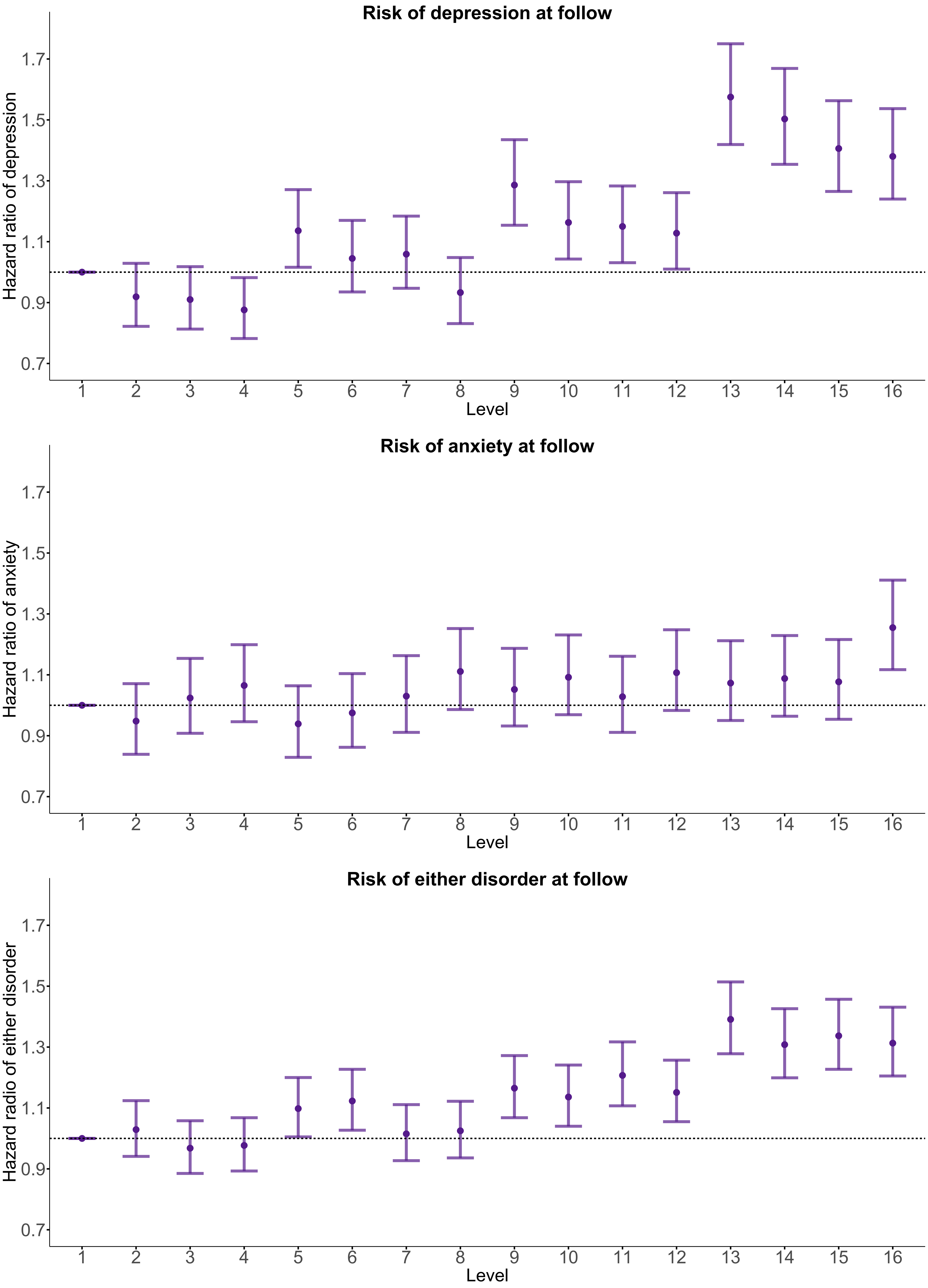
Risk of depression at follow
Hazard ratio of depression
1.7
1.5
1.3
1.1
0.9
0.7
1 2 3 4 5 6 7 8 9 10 11 12 13 14 15 16
Level
Risk of anxiety at follow
Hazard ratio of anxiety
1.7
1.5
1.3
1.1
0.9
0.7
1 2 3 4 5 6 7 8 9 10 11 12 13 14 15 16
Level
Risk of either disorder at follow
Hazard radio of either disorder
1.7
1.5
1.3
1.1
0.9
0.7
1 2 3 4 5 6 7 8 9 10 11 12 13 14 15 16
Level
